# Brain antibodies in the cortex and blood of people with schizophrenia and controls

**DOI:** 10.1038/tp.2017.134

**Published:** 2017-08-08

**Authors:** L J Glass, D Sinclair, D Boerrigter, K Naude, S J Fung, D Brown, V S Catts, P Tooney, M O'Donnell, R Lenroot, C Galletly, D Liu, T W Weickert, C Shannon Weickert

**Affiliations:** 1 Schizophrenia Research Laboratory, Sydney, NSW, Australia; 2 Neuroscience Research Australia, Sydney, NSW, Australia; 3 School of Psychiatry, University of New South Wales, Sydney, NSW, Australia; 4 St Vincent’s Centre for Applied Medical Research, St Vincent's Hospital, Sydney, NSW, Australia; 5 ICPMR, Westmead Hospital, Westmead, NSW, Australia; 6 School of Biomedical Sciences and Pharmacy, University of Newcastle, Newcastle, NSW, Australia; 7 Discipline of Psychiatry, Adelaide University, Adelaide, SA, Australia; 8 Ramsay Health Care, Adelaide, SA, Australia; 9 Northern Adelaide Local Health Network, Adelaide, SA, Australia

## Abstract

The immune system is implicated in the pathogenesis of schizophrenia, with elevated proinflammatory cytokine mRNAs found in the brains of ~40% of individuals with the disorder. However, it is not clear if antibodies (specifically immunoglobulin-γ (IgG)) can be found in the brain of people with schizophrenia and if their abundance relates to brain inflammatory cytokine mRNA levels. Therefore, we investigated the localization and abundance of IgG in the frontal cortex of people with schizophrenia and controls, and the impact of proinflammatory cytokine status on IgG abundance in these groups. Brain IgGs were detected surrounding blood vessels in the human and non-human primate frontal cortex by immunohistochemistry. IgG levels did not differ significantly between schizophrenia cases and controls, or between schizophrenia cases in ‘high’ and ‘low’ proinflammatory cytokine subgroups. Consistent with the existence of IgG in the parenchyma of human brain, mRNA and protein of the IgG transporter (FcGRT) were present in the brain, and did not differ according to diagnosis or inflammatory status. Finally, brain-reactive antibody presence and abundance was investigated in the blood of living people. The plasma of living schizophrenia patients and healthy controls contained antibodies that displayed positive binding to Rhesus macaque cerebellar tissue, and the abundance of these antibodies was significantly lower in patients than controls. These findings suggest that antibodies in the brain and brain-reactive antibodies in the blood are present under normal circumstances.

## Introduction

There is increasing evidence of immune abnormalities in people with schizophrenia. In the blood, increased concentration of cytokines, particularly interferon (IFN)-γ, interleukin (IL)-1β, soluble IL-2 receptor (sIL-2R), IL-6, IL-12, transforming growth factor (TGF)-β and tumor necrosis factor (TNF)-α, are found in people with schizophrenia when compared to controls.^1, 2^ In the brain, specifically dorsolateral prefrontal cortex (DLPFC), increased mRNA expression of IL-6, IL-1β and IL-8 cytokines can be found in some people with schizophrenia.^3, 4, 5, 6^ Transcript levels of various immune regulators and their chaperone proteins are also altered in the prefrontal cortex of subjects with schizophrenia.^7, 8^ Antipsychotic medications can have immunomodulatory effects,^9, 10, 11^ often lowering cytokine levels in addition to alleviating positive symptoms of schizophrenia. However, blood levels of IL-1β, IL-6, IL-12, IFN-γ, TNF-α, sIL-2R and TGF-β have been found to be elevated in unmedicated first-episode psychosis^1, 9, 12^ and chronically medicated patients,^13, 14^ indicating that antipsychotic treatment neither solely explains, nor completely remediates, immune activation in schizophrenia.

To date, it is unclear whether antibodies play a role in immune dysregulation in schizophrenia. The T-cell-produced cytokines activate B cells to switch from producing weakly binding immunoglobulin-μ to the highly specified immunoglobulin-γ (IgG). Playing an integral part in the secondary immune response, IgG antibodies bind complement, facilitate phagocytosis through opsonization, and direct cytotoxic activities of natural killer cells.^15^ In peripheral blood, elevated B-cell and reduced T-cell populations have been found in schizophrenia.^16, 17, 18^ In fact, mature B cells numbers appear to normalize in some schizophrenia patients whose clinical state has improved with antipsychotic treatment.^17, 19^ These observations suggest that immune dysregulation in schizophrenia may include an underlying component of B-cell pathology.

Antibodies in schizophrenia pertaining to brain pathology are likely to recognize brain antigens (brain-reactive) and should be present within the brain itself. Brain-reactive antibodies are known to be present in the blood in health^20^ and psychiatric disease,^20, 21, 22, 23, 24, 25, 26^ and may reflect antibody-related immune pathology in schizophrenia. Antibodies from blood have been shown to bind to monkey and human brain tissue antigens.^21, 22^ More specifically, antibodies targeting *N*-methyl-d-aspartate receptors (NMDAR) are found in the cerebrospinal fluid and serum of people with NMDAR encephalitis, who exhibit schizophrenia-like symptoms including psychosis and cognitive impairments,^27, 28^ and in some people experiencing first-episode psychosis.^29^ In people with schizophrenia, there are serum antibodies targeting other neurotransmitter receptors (for example, muscarinic cholinergic receptor 1, opioid receptor-μ and serotonin receptor-1A receptors),^23, 24, 25^ heat-shock proteins^30, 31, 32^ and glyceraldehyde 3-phosphate dehydrogenase^26^ (GAPDH). However, whether the human brain has an appreciable amount of IgGs, and whether their abundance is altered in schizophrenia, is unknown.

The presence of IgGs in the brain, particularly the healthy brain, is more plausible if mechanisms exist in the brain to allow their influx and efflux. The Fc fragment neonatal receptor (FcRN) is found in the choroid plexus and microvascular endothelial cells^33^ and facilitates the transit of IgG across the luminal surface.^34^ It is composed of a heavy chain, Fc region of IgG-targeting receptor transporter (FcGRT), and a light chain, β-2-microglobulin.^35^ Acting in a pH-dependent manner, FcRN binds to IgG at an acidic pH and releases it at a neutral pH.^36, 37^ As a result, the expression of FcGRT may influence the abundance of antibodies in the brain in psychiatric disease.

Therefore, in this study we aimed to (1) determine the presence of IgGs in the postmortem brains of people with schizophrenia and controls with a focus on the DLPFC and orbitofrontal cortex (OFC); (2) compare IgG levels between people with schizophrenia and controls previously categorised as ‘high inflammation’ or ‘low inflammation’ based on proinflammatory cytokine levels;^3, 4, 5^ (3) compare the abundance of FcGRT mRNA and protein between the aforementioned groups; and (4) assess the prevalence of brain-reactive antibodies in the plasma of a cohort of living schizophrenia patients and controls.

## Materials and methods

### Tissue and blood

#### Human brain tissue

Post-mortem brain tissue samples (*n*=79 individuals) were obtained from the New South Wales Tissue Resource Centre. OFC tissue from the medial gyrus rectus to include BA11 (between the branches of the orbital sulcus)^38^ was obtained from individuals with schizophrenia (*n*=38) and controls (*n*=38) and was cryostat sectioned in the coronal plane with sections mounted onto glass slides (Supplementary Table S1). Chunks of pulverized DLPFC gray matter (40 mg) from BA46 (middle frontal gyrus)^39^ was obtained from individuals with schizophrenia (*n*=37) and controls (*n*=37; Supplementary Table S2). Sample sizes were chosen based on variance observed in previous studies, with a sample size of *n*=74 expected to have >80% power to detect an effect size *d*=0.25.^39, 40^ This study was carried out in accordance with the latest version of the Declaration of Helsinki after review by the Human Research Ethics Committee at the UNSW (HREC #12435).

#### Rhesus macaque brain tissue

Fresh frozen frontal perfused rhesus macaque (*Macaca mulatta*, *n*=7) cortex (containing the principal sulcus) was cryostat sectioned in the coronal plane. All research procedures with non-human primates from the National Institutes of Mental Health (NIMH, USA) and were carried out in adherence to the regulations of the U.S. Animal Welfare Act (USDA, 1990) and Public Health Service Policies (PHS, 2002), in accordance with the ILAR ‘Guide for the Care and Use of Laboratory Animals’, and are described in Fung *et al.*^41^ The study was performed under an Animal Study Protocol approved by the NIMH Animal Care and Use Committee.

#### Human serum and plasma

Samples were obtained from a living cohort of controls (*n*=73) and people with schizophrenia (*n*=94)^42^ (Supplementary Table S3). Patients were matched to healthy controls based on gender and age within 5 years. Informed consent was obtained in accordance with a protocol approved by the University of New South Wales (UNSW) and the South Eastern Sydney, Illawarra Area Health Service Human Research Ethics Committees (HREC #07259, HREC #07121), and the Queen Elizabeth Hospital Human Ethics Committee (SA; HREC # 8222 6841). To prepare serum, whole blood was collected in SST tubes (BD Biosciences, Franklin Lakes, NJ, USA), incubated at room temperature (RT) for 30 min, centrifuged at 2000 *g* for 5 min at 4 °C. To prepare plasma, whole blood was collected in EDTA tubes (BD Biosciences), centrifuged at 1200 *g* for 15 min at 4 °C. The resulting serum, or plasma, was transferred to low binding tubes and stored at −80 °C.

### Immunohistochemistry

#### Immunohistochemistry to detect endogenous IgG in human OFC and rhesus macaque PFC

Human postmortem OFC sections from schizophrenia cases and controls, or rhesus macaque PFC, were thawed (RT for 20 min), fixed with 4% paraformaldehyde, washed (3 × PBS, 5 min) and submerged in 3:1 100% methanol in 3% H_2_O_2_ for 20 min at RT to block endogenous peroxidases. For human OFC, tissue was washed and blocked overnight with 10% normal rabbit serum (S-5000, Vector Laboratories, Peterborough, UK). For rhesus macaque PFC, tissue was blocked for 1 h at RT with 10% normal goat serum (S-1000, Vector Laboratories) and incubated overnight with mouse anti-monkey IgG primary (1:500, 4700-01, Southern Biotech, Birmingham, AL, USA). The next day, tissue was washed as above and incubated for 1 h at RT with (for human OFC) biotinylated rabbit anti-human IgG ‘secondary’ antibody preabsorbed against mouse (1:200, Ab7159, Abcam, Cambridge, UK) or (for rhesus macaque PFC) biotinylated goat anti-mouse IgG (1:500, BA9200, Vector Laboratories). After washing again, the tissue was incubated for 1 h at RT with avidin-biotin-peroxidase complex (VectaStain ABC kit, PK-4000, Vector Laboratories). Then 3’3-diaminobenzidine (DAB, 12 mm final concentration in PBS with 3% H_2_O_2_) was applied to the tissue for 3 min, before Nissl counterstaining (3 min exposure with 0.002% thionin). Images were taken with a Nikon Eclipse i80 microscope (Nikon, Tokyo, Japan) using a × 20 objective, and with contrast enhanced with ImageJ (v1.50.e, NIH, Bethesda, MD, USA).

#### Human OFC fluorescent immunohistochemistry

Fresh frozen OFC sections from people with schizophrenia (*n*=9) and healthy controls (*n*=9) were fixed, washed and blocked with 10% normal goat serum and 10% donkey serum (Jackson Immunoresearch Laboratories, Baltimore, MD, USA) in diluent for 1 h at RT. Tissue was then incubated overnight at 4 °C with rabbit anti-collagen IV (1:5000, AB6586, Abcam), biotinylated goat anti-human IgG (1:200, AB97168, Abcam), and mouse anti-neuronal nuclei (1:1000, mAB377, Chemicon International, Australia) primary antibodies. The following day tissue was washed as above and incubated in the dark with goat anti-rabbit IgG AlexaFluor 405 preabsorbed against chicken, cow, horse, human, mouse, pig and rat (1:500, AB175654, Abcam), streptavidin AlexaFluor 647 (1:1000, S21374, Life Technologies, Eugene, OR, USA) and donkey anti-mouse IgG AlexaFluor 488 preabsorbed against chicken, cow, goat, human, rabbit, rat, and sheep (1:500, AB150109, Abcam) for 1 h at 4 °C. Tissue was washed twice in PBS and then for 5 min in 10 μM acridine orange hemi(zinc chloride) (Ab146348, Abcam) in PBS at RT. Slides were washed twice in 5 mM cupric sulfate and 50 mM ammonium acetate solution for 15 min at RT to quench autofluorescence. Tissue was then mounted with fluorescent-friendly immersion oil (Citifluor AF1 anti-fadent, ProSciTech, Thuringowa, QLD, Australia) and slide edges were sealed with nail polish. Z-stack spectral images were captured using a Nikon Eclipse 90i laser-scanning microscope and subjected to blind unmixing. Images were taken at × 40 and contrast and brightness enhanced using ImageJ.

#### Immunohistochemistry using human serum from living people as a ‘primary’ antibody

Pooled serum from live individuals with schizophrenia (*n*=10), or pooled serum from healthy controls (*n*=10) was diluted (1:150, 1:300, 1:700) to be used as ‘primary’ antibodies. Perfused Rhesus macaque cerebellar sections^43^ were used to so that brain-reactive IgG in the blood could be detected without IgG in the blood vessels and/or brain confounding the results. Sections were treated the same as the Rhesus macaque PFC above except that pooled serum was used as the ‘primary’ antibody and the secondary antibody was goat anti-human (1:250).

### Quantitative real-time PCR

RNA was extracted and cDNA synthesized from DLPFC tissue of people with schizophrenia (*n*=37) and healthy controls (*n*=37) as previously described in Weickert *et al.*^39^ Transcript levels were measured by qPCR using Applied Biosystems’ Prism 7900HT Real-time PCR system (Foster City, CA, USA). A pre-designed Taqman gene expression assay from Applied Biosystems (Foster City, CA, USA) was used for FCGRT (Hs01108967_m1), normalized to the geometric mean of four housekeeper genes; β-actin (Hs99999903_m1), GAPDH (Hs99999905_m1), TATA box binding protein (Hs00427620_m1), and ubiquitin C (Hs00824723_m1) that did not vary in expression between diagnostic groups.^39^

### Western blotting

#### Human DLPFC western blotting

For endogenous IgG detection, DLPFC tissue from schizophrenia cases (*n*=37) and controls (*n*=37) was homogenized in buffer (50% 0.1 m Tris Buffer pH7.5, 50% glycerol, protease inhibitor cocktail 1:100 and aprotinin 1:1 600) and 10 μg of each sample electrophoresed for 75 min at 120 V on a 10% bis-tris polyacrylamide gels alongside a molecular weight ladder (Precision Plus, BioRad Laboratories, Hercules, CA, USA) and a pooled internal control (IC) sample. Proteins were transferred onto nitrocellulose membranes (BioRad) at 100 V for 2 h, and then blocked for 2 h at 4 °C in 5% skim milk in Tris-buffered saline (TBS) containing 0.1% Tween-20 (TBST). As this assay was to detect IgG in the brain blots were left in TBS at 4 °C for 1–2 nights without a primary. Blots were incubated with horse radish peroxidase (HRP) conjugated goat anti-human IgG secondary antibody (1:5000; #PA1-28829, Pierce antibodies, Rockford, IL, USA) for 1 h at RT. Immunoreactive bands were detected using the enhanced chemiluminesence (ECL) detection kit (Amersham Biosciences, Piscataway, NJ, USA) and were exposed to ECL Hyperfilm (Amersham Biosciences). Membranes were then stripped (stripping buffer 25 mM glycine, 1.5% SDS, pH2.0) and reprobed with mouse anti-β-actin primary antibody (1:10 000; MAB1501, Merck Millipore, Billerica, MA, USA) and HRP conjugated goat anti-mouse secondary antibody (1:5000; AP124P, Merck Millipore). Immunoreactive band intensities were normalized to the intensity of the β-actin band in the same lane and the IC (27.75% interblot variability) from the same gel. Samples were run in duplicate, in separate experimental runs and averaged, and quantified with Image J.

FcGRT protein was quantified by western blot as described above but using Odyssey detection (LI-COR Biosciences, Lincoln, NE, USA). Proteins were transferred onto Immobolin-FL PVDF membrane (IPFL20200, Merck Millipore), blocked with LI-COR TBS blocking buffer and probed with rabbit anti-FcGRT IgG primary antibody (H-274; 1:200, sc-66892, Santa Cruz, Dallas, TX, USA) and the same mouse anti-β-actin as above, and paired with IRDye 800 CW donkey anti-rabbit IgG (1:15 000, 925-32213, LI-COR) and IRDye 680 RD donkey anti-mouse (1:10 000, 925-68072, LI-COR) secondary antibodies respectively. Bands were visualized using the Odyssey scanner (LI-COR) and quantified with Image Studio Lite software (LI-COR).

#### Western blotting using pooled human serum as the ‘primary’ antibody

Brain-reactive IgG in the serum of living schizophrenia cases (*n*=10) and controls (*n*=10) was detected by Western blot using the ECL detection kit (Amersham Biosciences) and 10 μg of protein from homogenized rhesus macaque cerebellar tissue. Blots were incubated overnight with the serum ‘primary’ antibody (1:200 1% skim milk in TBST). The following day, blots were incubated with HRP conjugated goat anti-human secondary (1:5000; #PA1-28829, Pierce) before ECL detection as above.

#### Indirect immunofluorescence for plasma brain-reactive antibodies

Plasma samples from living people with schizophrenia (*n*=94) and living healthy controls (*n*=72) were diluted 1:10 in PBS containing 0.1% Tween-20 (PBST) and applied to BIOCHIP Slides (EuroImmun, Lübeck, Germany) using the titerplane technique for 30 min at RT. BIOCHIP Slides contained 10 reaction fields each with 4 substrates, one of which was primate cerebellum. BIOCHIP Slides were rinsed and then immersed in PBST for 5 min before incubation with fluorescein labeled anti-human globulin for 30 min at RT. BIOCHIP Slides were rinsed and immersed again in PBST for 5 min and then mounted so that reaction fields were embedded in glycerol/PBS, as per manufacturer’s instructions. A Nikon Eclipse 90i laser-scanning microscope with × 20 objective lens and NIS Elements software were used to examine and image BIOCHIP Slides. An IC (reaction field with pooled plasma from 10 controls and 10 schizophrenia patients) and manufacturer supplied negative control (1:10 in PBST) were included for each round of analysis. Brain-reactive IgGs were considered present in the plasma if the pixel intensity of primate cerebellar staining was greater than two standard deviations from the mean pixel intensity of the negative controls.

### Data analyses

All analyses were performed using the Statistical Package for Social Sciences (version 22, IBM, Armonk, NY, USA) or GraphPad Prism (version 6.04 La Jolla, CA, USA). To achieve normal distribution average IgG levels were square root transformed (postmortem brain and plasma cohorts), and brain FcGRT mRNA/protein levels were log transformed. Grubbs tests yielded no outliers for brain IgG, FcGRT protein or FcGRT mRNA or blood IgG. Inflammatory subgroups were previously classified based on the mRNA expression of four inflammatory cytokines identified through two-step recursive clustering as described in Fillman *et al.*^3^ Due to low sample size (*n*=4) the control high inflammation subgroup was excluded for the analysis according to inflammatory subgroups. Demographic variables (age at death, freezer months, pH, postmortem interval (PMI) and mRNA integrity number) were included as covariates in analyses of group differences if they were significantly correlated with the variable of interest, as determined by Pearson’s correlation. In the absence of such correlations, Student’s t-tests or one-way ANOVAs were used, followed by Fischer’s LSD *post hoc* tests if *P*<0.05. Levene’s test was used to determine homogeneity of variance between groups and when required the statistic adjusted for unequal variances (*n*=1 test) reported. To determine whether inflammatory history in the week before death, or cause of death, influenced IgG abundance in the cortex of schizophrenia cases or controls, factorial ANOVAs with diagnosis and inflammatory history (yes/no) or cause of death (cardiac complications—yes/no) were used. For cause of death, individuals who died of cardiac complications were compared to those who died of other causes because sample sizes for other causes of death (respiratory (*n*=3), suicide (*n*=0), other (*n*=4)) within diagnostic groups were too small for meaningful comparison. Chi-squared test was used to compare the incidence of plasma brain-reactive IgG positivity in schizophrenia and control groups.

## Results

### IgG is present in the orbitofrontal cortex of controls and people with schizophrenia

**Figure 1:**
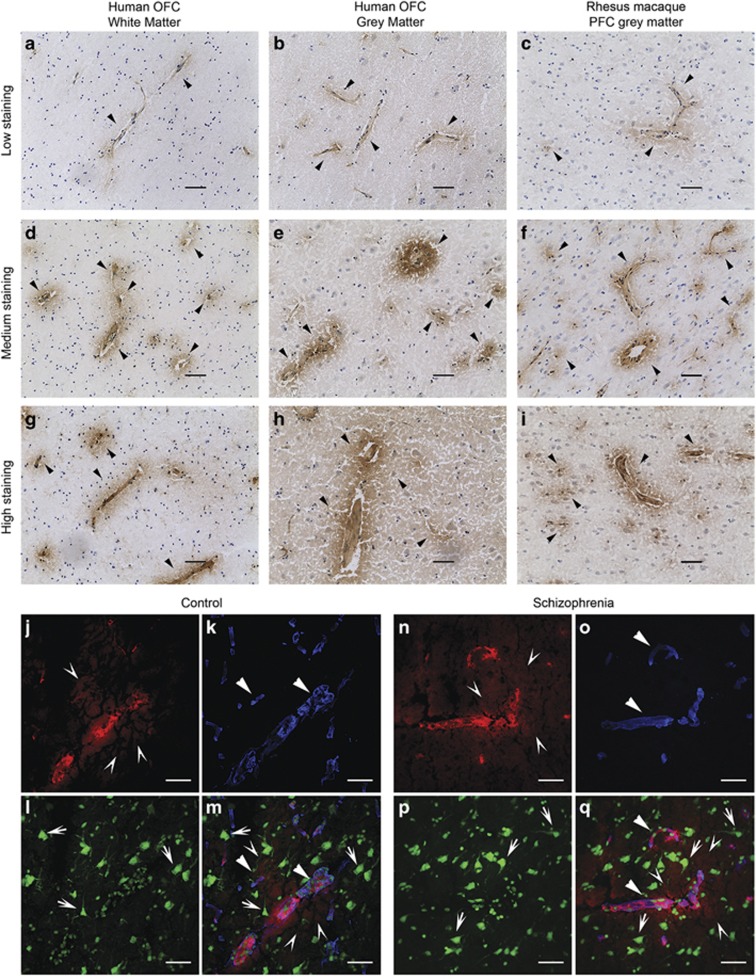
Endogenous IgG antibodies are present in low (**a**, **b**), medium (**d**, **e**) and high levels of (**g**, **h**) intensity in both the white (**a**, **d**, **g**) and gray matter (**b**, **e** and **h**) of the orbitofrontal cortex (OFC) of people with schizophrenia (rabbit anti-human IgG (**a**, **b**, **d**, and **e**)) and healthy controls (anti-human IgG (**g**, **h**)). No obvious qualitative differences were seen between diagnostic groups. IgG antibodies were also detected in the prefrontal cortex (PFC) of perfused Rhesus macaques (mouse anti-monkey IgG (**c**, **f**, and **i**)). Arrowheads indicate the extent of IgG signal surrounding blood vessels (closed arrowheads **a - i**). Images taken with a 20x objective. Colocalization of endogenous IgG (goat anti-human IgG; open arrows, red (**j** and **n**) pink (**m** and **q**),) surrounding blood vessels (rabbit anti-collagen IV; closed arrowheads; blue (**k**, **m**, **o**, and **q**)) and neurons (mouse anti-NeuN; arrow demarcate some cell bodies, green (**l**, **m**, **p**, and **q**)) in the orbitofrontal cortex of healthy controls (**j**, **k**, **l**, and **m**) and people with schizophrenia (**n**, **o**, **p**, and **q**). Despite no colocalization of endogenous IgG with neuronal cell bodies, the diffusing halo (open arrows) from blood vessels overlaps with processes of some neurons. Scale bars are 50 μm. Images were subjected to blind spectral unmixing and taken with a 40x objective.

IgG was detected by immunohistochemistry in the OFC of people with schizophrenia (*n*=38, Figure 1a, b, d and e) and controls (*n*=38, Figure 1g and h) as indicated by a diffuse brown DAB reaction product. A darker halo of brown reaction product was often visible surrounding many blood vessels (Figure 1a–i arrowheads). The immunoreactivity appeared to radiate outwards from the blood vessel. The degree of signal extension into the brain parenchyma varied between individuals irrespective of diagnosis and even from blood vessel to blood vessel within the same brain (Figure 1a–i).

To exclude the possibility that IgG immunoreactivity in the human OFC was artifact arising from tissue degradation or diffusion of residual blood components into the tissue with prolonged PMI, we performed the same immunohistochemistry using saline perfused PFC of Rhesus macaques and an anti-rhesus IgG antibody (*n*=7, Figure 1c, f, and i). As with the human OFC, the DAB signal from the IgG immunoreactivity was found in and surrounding various blood vessels, dissipating into the brain parenchyma. Immunoreactivity was absent in the “no secondary” human, and primate no primary, control sections (Supplementary Figure S1).

### IgG associates mainly with blood vessels

Immunoreactivity (red) indicating presence of endogenous IgGs (Figure 1j and n) was closely associated with collagen IV-positive (blue) blood vessels (Figure 1k and o) in both controls (*n*=9) and people with schizophrenia (*n*=9, Figure 1j and m and n-q respectively). A diffuse halo of IgG staining radiated from some, but not all, blood vessels. IgG signal varied in intensity in the parenchyma between individuals. Cell bodies (green) of neurons (Figure 1l and p), did not appear to be directly associated with IgG signal, however the halo of IgG appeared to overlap with the processes of some neurons. These processes were often adjacent to IgG positive blood vessels. None of the no primary control slides had immunoreactive signal (Supplementary Figure S2).

### IgG levels in human prefrontal cortex do not differ significantly between diagnostic or inflammatory groups

An immunoreactive band at the weight consistent with that of the IgG heavy chain (50kDa) was detected in all humans tested (*n*=74). The abundance of IgG was not different when comparing schizophrenia cases and controls (*t*(72)=−0.991, *P*=0.325), or those with high inflammation (*n*=18) compared with low inflammation regardless of diagnosis (*n*=56; *t*(72)=-1.541, *P*=0.128). Similarly, comparisons between controls (*n*=33), high inflammation schizophrenia cases (*n*=14), and low inflammation schizophrenia cases (*n*=23) were not significant (one-way ANOVA: *F*(2,67)=1.767, *P*=0.179). There was no main effect of history of inflammation before death (Supplementary Table S2) on levels of IgG (factorial ANOVA, F(1,70)=0.57, *P*=0.45), nor an interaction of history of inflammation before death with diagnosis (*P*=0.74). Similarly, there was no main effect of cause of death (cardiac complications) on levels of IgG (factorial ANOVA, F(1,70)=0.001, *P*=0.97), nor an interaction of cause of death with diagnosis (*P*=0.94).

### FcGRT levels in human dorsolateral prefrontal cortex do not differ between diagnostic or inflammatory groups

**Figure 2:**
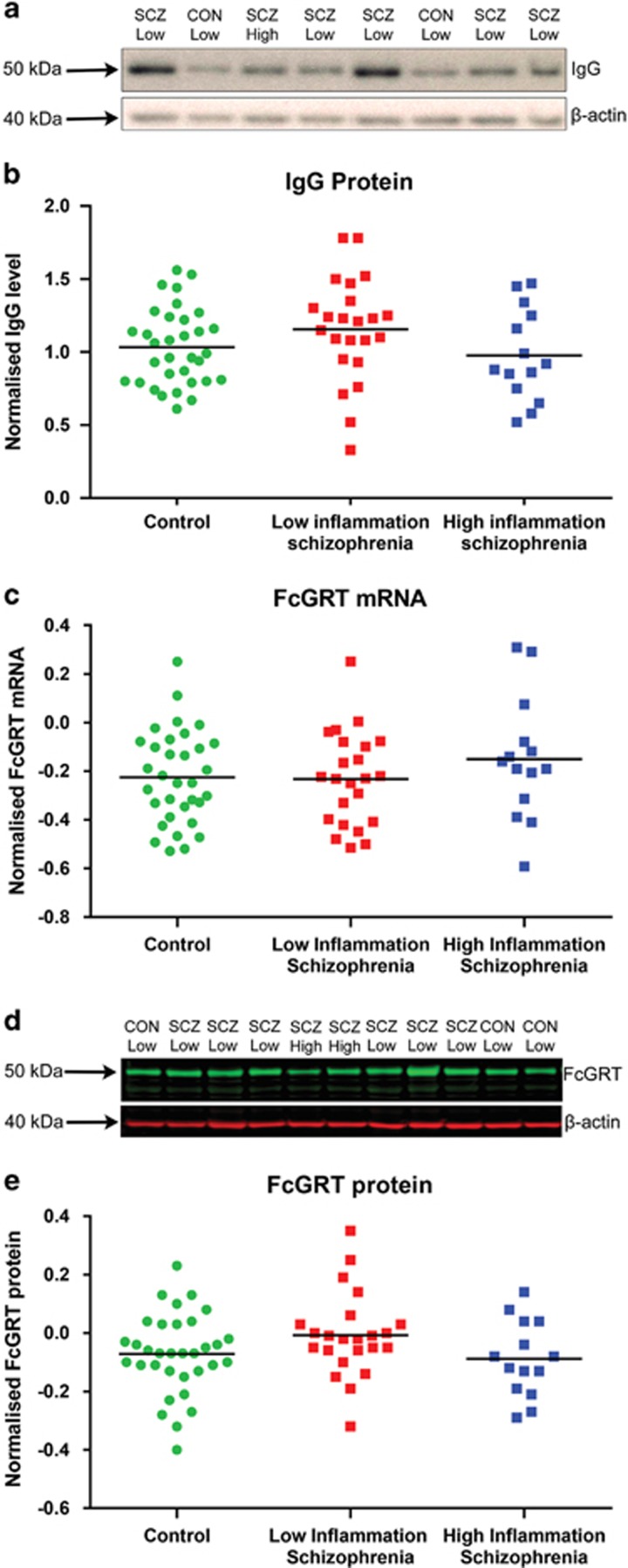
An anti-IgG immunoreactive band of 50 kDa (**a**) was found using western blotting on dorsolateral prefrontal cortex homogenate of all humans studied (*n*=74). The intensity of the IgG varied from one human brain to another while the level of β-actin (at 42 kDa) was of similar abundance. IgG abundance did not differ by diagnosis of inflammatory subgroup (**b**) Horizontal bars represent group means. Expression of FcGRT mRNA was comparable between high and low inflammation schizophrenia cases and controls (**c**) Representative western blot probed for FcGRT protein in the human DLPFC (**d**) Protein levels of FcGRT in the DLPFC did not differ between high inflammation schizophrenia cases, low inflammation schizophrenia cases or controls (**e**) Horizontal bars represent group means.

FcGRT mRNA (Figure 2c) and protein (Figure 2d and e) in the DLPFC were investigated by qPCR and western blotting respectively. FcGRT mRNA expression significantly correlated with PMI (*r*=−0.281, *P*=0.015), but not brain tissue pH. Normalized FcGRT mRNA levels did not differ between individuals with schizophrenia and controls (ANCOVA, covarying for PMI, *F*(1,71)=0.213, *P*=0.646), between high inflammation and low inflammation groups overall (ANCOVA, covarying for PMI, *F*(1,71)=2.985, *P*=0.88) or among high inflammation schizophrenia cases, low inflammation schizophrenia cases and controls (Figure 2c; ANCOVA, covarying for PMI, *F*(2,66)=0.745, *P*=0.478].

Probing for FcGRT protein by western blot, we detected a prominent immunoreactive band at approximately 50 kDa, slightly larger than the expected molecular weight of FcGRT at 40 kDa (Figure 2d). Intensity of this FcGRT immunoreactive band did not correlate with any demographic variables, and the intensity of FcGRT (FcGRT/ β-actin) did not differ between diagnostic groups (*t*(72)=−1.43, *P*=0.159) or inflammatory groups (*t*(72)=0.99, *P*=0.32). We did not detect a significant difference in FcGRT protein levels among high inflammation schizophrenia cases, low inflammation schizophrenia cases and controls (Figure 2e; *F*(2,67)=0.93, *P*=0.40).

### Brain-reactive IgG are present in the serum of living schizophrenia patients and living healthy controls

**Figure 3:**
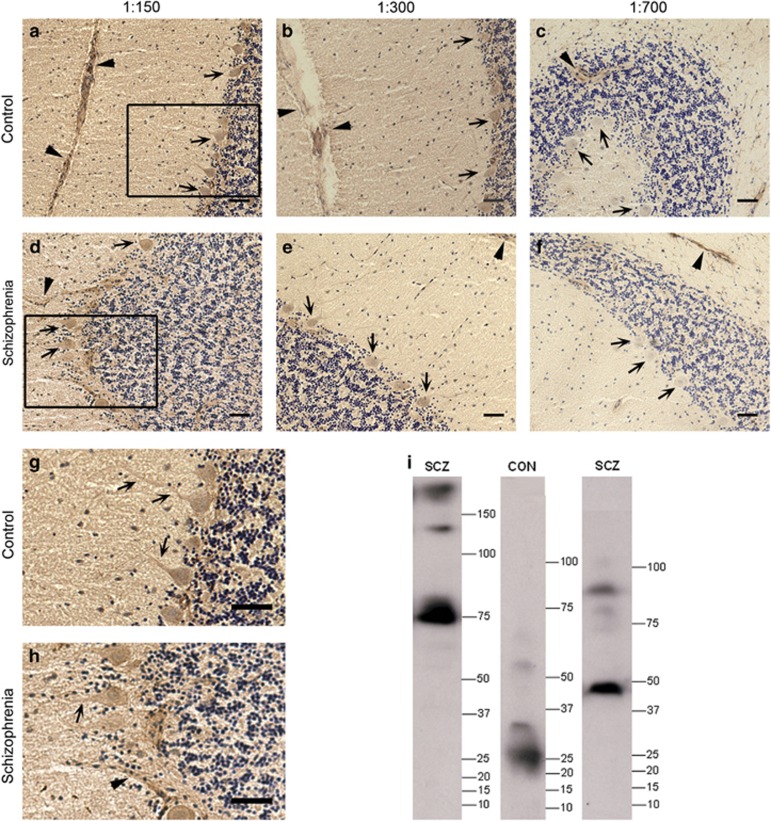
Brain-reactive IgG was identified in the serum of healthy controls and people with schizophrenia. Immunohistochemistry using pooled human serum from controls as the primary antibody on rhesus macaque cerebellar tissue sections (**a-c**). Immunohistochemistry as above, using pooled serum from people with schizophrenia on rhesus macaque cerebellar sections (**d–f**). Serial dilutions of serum are as indicated in the above images (**a**, **d**: 1:150; **b**, **e**: 1:300; **c**, **f**: 1:700). Structures which have IgG-reactive brain antigens are stained brown. Nissl stained nuclei are blue. Filled arrowheads indicate blood vessels. Arrows indicate Purkinje neurons. Enlargement of boxes in 3a and 3b (**g**, **h**). Scale bars are 50 μm. Western blot of protein from an adolescent rhesus macaque cerebellum using serum from two representative schizophrenia patients and one control as primary antibodies (**i**). Immunoreactive bands indicate a unique array of proteins targeted by serum IgGs for each individual. CON, control; SCZ, chizophrenia.

Brain-reactive IgG were detected using pooled serum samples from a cohort of living healthy controls (Figure 3a and c and g) and living people with schizophrenia (Figure 3d–f and h) as a primary antibody to Rhesus macaque cerebellum sections processed for DAB IHC. The DAB signal product decreased in a serum concentration-dependent manner (Figure 3a–f and h). Immunoreactivity observed at a 1:700 serum dilution (Figure 3c and f) was indistinguishable from the control slide (Supplementary Figure S3). Purkinje neurons apical dendrites were visible at higher serum concentrations, 1:150 (Figure 3a and d, arrows in Figure 3g and h) and 1:300 (Figure 3b and e). Dendrites were not always distinguishable due to the diffuse molecular layer staining. The Purkinje neuron cell bodies (Figure 3a–f arrows) were visible in all sections. Consistent staining of blood vessels was seen in all sections (Figure 3a–f arrowheads) including the no serum control (Supplementary Figure S3). Cross reactivity of monkey tissue with the anti-human secondary antibody alone was also evident in light brown fibrous staining throughout the tissue (Supplementary Figure S3). We did not find any qualitative difference in staining intensity obtained with control (Figure 3a and c and g) or patient (Figure 3d–f and h) serum.

### Brain-reactive serum IgG from living people recognize unique proteins

A western blot in which Rhesus macaque cerebellar protein was probed with serum from individual controls and schizophrenia cases confirmed the presence of brain-reactive IgG in serum from living people. For each serum sample, multiple immunoreactive bands of molecular weights from 25 to >150 kDa were identified (Figure 3i). Immunoreactive bands recognized by serum from each individual displayed a unique pattern and intensity.

### Brain-reactive IgGs are present in the plasma from living people and differ between people with schizophrenia and controls

**Figure 4:**
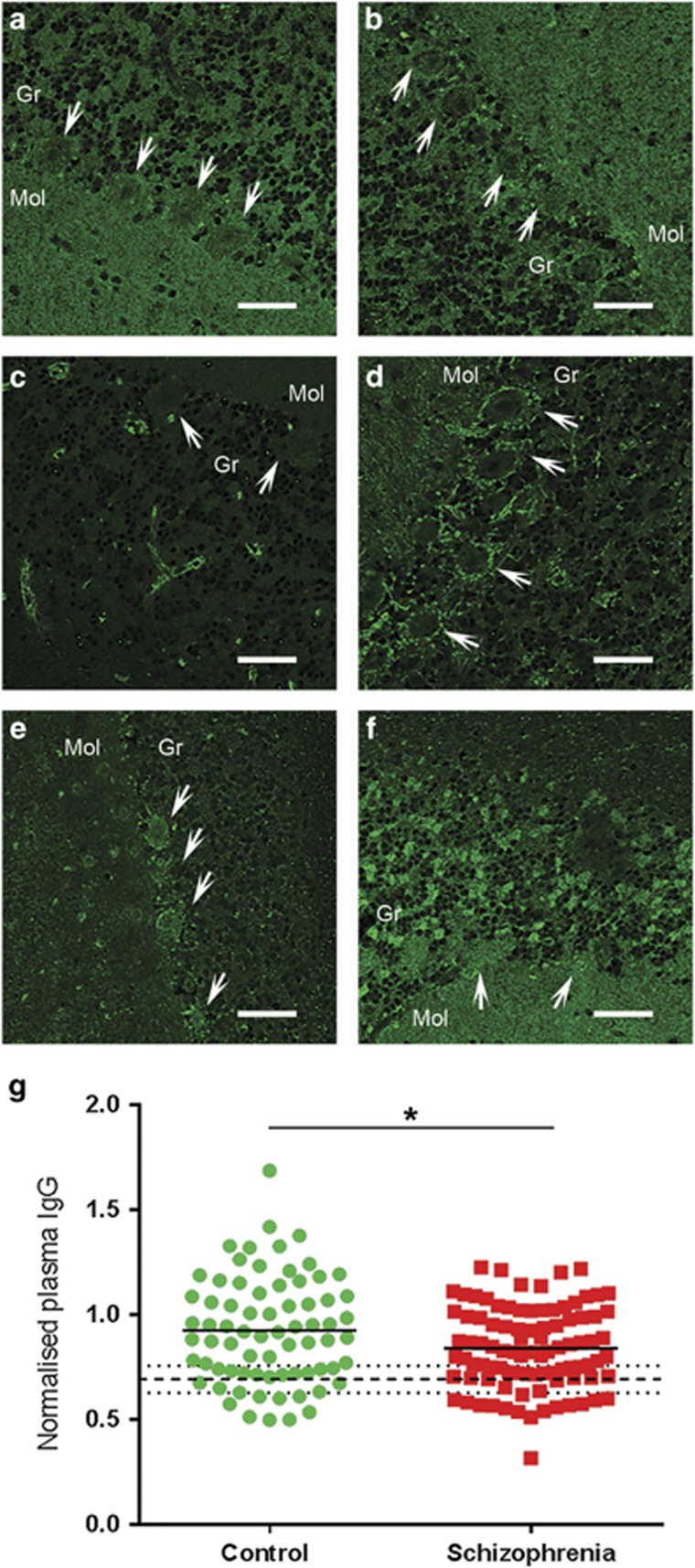
Plasma brain-reactive antibodies from a live patient cohort of people with schizophrenia (*n*=94) and controls (*n*=72) resulted in six different patterns of fluorescence (green) when applied to the primate cerebellar tissue of the Euroimmun Indirect Immunofluorescence Test: equivalent intensity in Purkinje neurons and molecular and granular layers (29%, 48/166) (**a**) low Purkinje neuron intensity (34%, 56/166) (**b**), distinctive blood vessels (6%, 10/166) (**c**), bright ring around Purkinkje neurons with fibers throughout (14%, 23/166) (**d**), bright Purkinje neurons with punctate molecular layer cells (10%, 17/166) (**e**) and granular layer cells and bright molecular layer (7%, 12/166) (**f**). Images taken at 20x magnification. Scale bars are 50μm. Antibody fluorescence intensity was lower in people with schizophrenia than controls, *t*(128.6)=−2.377, *P*=0.019 when adjusted for unequal variance (Levene’s test: *F*(1,164)=5.877, *P*=0.016) as denoted by asterisk (**g**). Solid horizontal bars represent group means. Dashed line indicates the average IgG intensity across the no plasma controls (**g**). Brain-reactive IgG was considered present if intensity of staining with plasma was greater than two standard deviations (dotted lines, **g**) above the average negative controls (dashed line; **g**). Arrows indicate Purkinje neurons. Mol, molecular layer; Gr, granular layer.

The Euroimmun Indirect Immunofluorescence Test was used to assess the abundance, and brain tissue binding, of brain-reactive IgG in plasma from people with schizophrenia and controls (*n*=166). Six non-mutually exclusive patterns of immunofluorescence—(1) ubiquitous, (2) Purkinje neurons, (3) blood vessel (4) fibrous, (5) punctate and (6) granular cells were evident in the primate cerebellum (Figure 4a–f, green signal), which were distinct from the pattern in the negative controls (Supplementary Figure S4). Homogenous staining across the molecular layer, granular layer and white matter was seen in most individuals (63%). In 29% of individuals, this staining was accompanied by Purkinje neuron signal (pattern 1, Figure 4a), while in 34% the Purkinje neurons appeared unlabeled (pattern 2, Figure 4b). A small number of individuals (6%) clearly show blood vessel immunoreactivity throughout the cerebellar tissue (pattern 3, Figure 4c). When fibrous staining was detected (pattern 4) it was most consistently found around the Purkinje neurons (14% of individuals; Figure 4d). Punctate molecular layer cell staining (pattern 5) was typically accompanied by intensely immunoreactive Purkinje cell bodies (10% of individuals; Figure 4e), whereas cellular staining in the granular layer (pattern 6) was consistent with darker Purkinje neurons (7% of individuals; Figure 4f).

There was no difference in the incidence of schizophrenia cases (62.77%, 59/94) and controls (68.06%, 49/72) which were clearly positive for brain-reactive antibodies (*χ*^2^=0.5, *P*=0.479). Using mean fluorescence pixel intensity as a semi-quantitative measure of IgG abundance, plasma from live schizophrenia patients contained slightly, but significantly, lower brain-reactive IgG levels than that of healthy living controls (Figure 4g; *t*(128.6)=−2.377, *P*=0.019 adjusting for unequal variance). IgG abundance did not correlate with plasma storage freezer time (*n*=166, *r*=0.103, *P*=0.189), or schizophrenia patient daily chlorpromazine equivalent dose (*n*=94, *r*=−0.59, *P*=0.572).

## Discussion

In this study, we found evidence for IgGs in the adult human cortex, particularly in diffuse patterns surrounding blood vessels but extending into brain parenchyma in both controls and people with schizophrenia. To our knowledge, we are the first to find evidence of, and to quantify, IgG within the normal human brain. In support of the human brain’s capacity for IgG movement across the blood brain barrier (BBB), we detected the IgG transporter (FcGRT), in brain at both the mRNA and protein levels. Contrary to our expectations, we failed to detect differences in the abundance of IgGs, FcGRT protein or FcGRT mRNA in the brains of people with schizophrenia compared with healthy controls. This suggests that IgG is normally present in, and actively effluxed from, the brain. Brain-reactive antibodies were also detected in serum of living people, and appeared to target a range of neural proteins. We did not observe differences in the incidence of plasma brain-reactive antibody-positivity between schizophrenia cases and controls from a cohort of living people, but semi-quantitative analysis suggested decreased levels of plasma brain-reactive IgGs in schizophrenia. Overall, we found that all individuals had IgG in the brain, with equivalent abundance in schizophrenia cases and controls, even when taking elevated proinflammatory cytokines^4^ into account.

One main limitation of our study is that many results are derived from postmortem brain (Figures 1, 2, 3, 4). However it is unlikely that the patterns of IgG in the brain observed in this study are artifacts associated with long PMI or the presence of residual blood components in cortical blood vessels. The diffuse pattern of IgG staining around the blood vessel in the cortex of humans was also seen in saline perfused rhesus macaques with very short PMI. This pattern is consistent with that observed in the saline perfused rodent brain,^44, 45^ as is the endothelial cell immunoreactivity we observed.^46^ These results from three mammalian species supports our use of immunohistochemical methods to investigate IgG in the brain of people with schizophrenia and controls, and indicate the suitability of monkeys and rodents as animal models for future studies.

IgG may enter the mammalian brain from the blood by crossing the BBB, or be produced by B cells that have transmigrated from the blood into the brain perivascular space.^28, 47, 48^ Transient hypertension from elevated adrenalin^49^ or stress-induced proinflammatory cytokines, which compromise endothelium junction integrity^50^ can also facilitate IgG entrance from the blood. Thus, we hypothesized that individuals with schizophrenia with increased peripheral and brain proinflammatory cytokines (high inflammation)^1, 4^ may have elevated endogenous brain IgG due to changes in the BBB.^51^ Contrary to this hypothesis, we were unable to detect a difference in brain IgG levels between high and low inflammation subgroups, or schizophrenia cases compared to controls. Instead, our results suggest that there is a quantifiable level of IgG in the healthy brain where they may contribute to normal functioning. Further testing of this idea could include assessing the impact of changing brain IgG levels on Fcγ-receptor abundance and function.

In our study, we found that over 60% of plasma from living people meet our criteria for positive immunostaining of brain tissue regardless of diagnosis, adding further support to the hypothesis that brain-reactive antibodies are ubiquitously found in human blood regardless of disease.^20, 52^ Such a high rate of antibody-positivity contrasts with prior reports of relatively lower incidence of brain-reactive antibodies in schizophrenia studies. An underlying cause of discrepancy may be that these studies focus on antibodies targeting certain pre-selected antigens in the blood^22, 24^ and therefore fail to analyze the plethora of other brain-reactive antibodies that may be present. That being said, unlike previous studies, ours did not rule out the binding of antibodies to antigens common to other organs. Future studies should consider preabsorbing samples to remove IgGs which target peripheral antigens^53, 54^ as this would circumvent this issue.

Although our study does not provide evidence of widespread antibody dysregulation in the schizophrenia brain, there is compelling evidence for deleterious effects caused by antibodies in the brain. Anti-NMDAR antibodies in NMDAR encephalitis and brain-reactive antibodies in neuropsychiatric SLE, play a causative role in psychiatric symptoms of both disorders.^55, 56^ Cultured cells treated with cerebrospinal fluid IgG from NMDAR encephalitis patients displayed decreased numbers of NMDARs on postsynaptic dendrites*in vitro*,^28^ but only impair behavior in mice with BBB dysfunction.^27^ Since comparable levels of anti-NMDAR antibodies are seen in the blood of schizophrenia patients and healthy controls,^27^ coincident BBB dysfunction may be required for antibody mediated pathology in schizophrenia. Importantly, while we did not detect widespread antibody-related abnormalities in the postmortem schizophrenia brain, or blood from living people with schizophrenia, our methods were inappropriate to investigate subtle dysregulation caused by antibodies targeting particular proteins.

Our work indicates that IgGs may be found in the brain under normal conditions. Measurement of the relative abundances of structurally different IgGs in the blood and brain of control cases would aid in determining the normal composition of the brain IgG population and provide initial insights into their actions. A focus on the origin, regulation, and function of brain IgG in future studies could aid in establishing the effects of IgG in the brain. Finally, the possibility of greater access to the brain of IgGs suggests that the psychiatric and neurological consequences of monoclonal antibody therapies may be more extensive than currently anticipated, and this requires further consideration.
