# Supplementary material for: Brain antibodies in the cortex and blood of people with schizophrenia and controls
Source: Transl Psychiatry. 2017 Aug 8;7(8):e1192–. doi: 10.1038/tp.2017.134 (PMC5611715; doi:10.1038/tp.2017.134)
Supplement: Supplementary Information [file tp2017134x1.docx]

**Supplementary information for “**Brain antibodies in the cortex and blood of people with schizophrenia and controls**”**

Corresponding author:

Professor Cynthia Shannon Weickert

Schizophrenia Research Laboratory

Neuroscience Research Australia

Barker Street

Randwick, NSW, Australia, 2031

Phone: +61 2 9399 1717

Email: [c.weickert@neura.edu.au](mailto:c.weickert@neura.edu.au)

This supplementary file includes the following tables; demographics of individuals in the OFC tissue collection (Supplementary Table S1), demographics of individuals in the DLPFC tissue collection (Supplementary Table S2), and demographics of individuals in the serum/plasma collection (Supplementary Table S3).

This supplementary file includes the following images; no secondary control or no primary DAB immunohistochemistry of human orbitofrontal cortex (Supplementary Figure S1a) and perfused Rhesus macaque prefrontal cortex (Supplementary Figure S1b) respectively, no primaries control for immunofluorescence of human orbitofrontal cortex (Supplementary Figure S2), no serum control for rhesus macaque incubated with human serum as the primary antibody DAB immunohistochemistry (Supplementary Figure S3), no serum control for Euroimmun indirect immunofluorescence primate cerebellum with human serum (Supplementary Figure S4).

**Supplementary Table S1: Demographics of individuals in the OFC tissue collection**

|  | **Control** | **Schizophrenia** |
| --- | --- | --- |
| N | 38 | 38 |
| Age in years (range) | 51.55 (18-78) | 52.05 (27-75) |
| Gender | 9F:29M | 13F:25M |
| Hemisphere | 23R:15L | 19R:19L |
| Tissue pH ± S.D | 6.63 ± 0.30 | 6.59 ± 0.30 |
| Postmortem interval (hours) ± S.D. | 24.66 ± 11.06 | 29.79 ± 12.84 |
| Freezer storage time (months) ± S.D. | 69.57 ± 42.73 | 78.80 ± 36.84 |
| RIN ± S.D. | 7.59 ± 0.83 | 7.51 ± 0.84 |
| Age (years) at onset (range) | - | 23.77 (14-40) |
| Duration of illness (years) ± S.D. | - | 27.58 ± 14.06 |
| Chlorpromazine median dose (mg) (range) | - | 575.16 (162.50-2362.50) |

R: right, L: left, F: female, M: male, S.D.: standard deviation

**Supplementary Table S2: Demographics of individuals in the DLPFC tissue collection**

|  | | **Control** | **Schizophrenia** |
| --- | --- | --- | --- |
| N | | 37 | 37 |
| Age in years (range) | | 51.1 (18-78) | 51.32 (27-75) |
| Gender | | 7F:30M | 13F:24M |
| Hemisphere | | 23R:14L | 17R:20L |
| Tissue pH ± S.D | | 6.67 ± 0.29 | 6.61 ± 0.30 |
| Postmortem interval (hours) ± S.D. | | 24.8 ± 10.97 | 28.45 ± 13.77 |
| Freezer storage time (months) ± S.D. | | 69.62 ± 42.71 | 78.89 ± 37.24 |
| RIN ± S.D. | | 7.30 ± 0.57 | 7.27 ± 0.58 |
| Age (years) at onset (range) | | - | 23.70 (14-40) |
| Duration of illness (years) ± S.D. | | - | 27.62 ± 13.82 |
| Chlorpromazine median dose (mg) (range) | | - | 691.64 (162.50-2362.50) |
| Inflammatory history during week prior to death (n) (%) | | 13/ (35.1) | 15 (40.5) |
| **Cause of death** | |  |  |
|  | Cardiac complications (n) (%) | 30 (81.1) | 18 (48.6) |
|  | Respiratory complications (n) (%) | 4 (10.8) | 2 (5.4) |
|  | Suicide (n) (%) | 0 (0) | 8 (21.6) |
|  | Other (n) (%) | 3 (8.11) | 9 (24.3) |

R: right, L: left, F: female, M: male, S.D.: standard deviation, %: percent. Evidence for an inflammatory history in the last week prior to death and cause of death were included in the medical records of all individuals studied. Details for each case are in Supplementary Table 1 of Fillman et al^1^.

**Supplementary Table S3: Demographics of individuals in the serum/plasma collection**

|  | **Control** | **Schizophrenia** |
| --- | --- | --- |
| N | 72 | 94 |
| Age in years- mean (range) | 32 (20-49) | 36 (20-51) |
| Sex | 34F, 36M | 37F, 56M |
| Age (years) at onset (range) | - | 22.8 ± 5.5 |
| Duration of illness (years) ± S.D. | - | 13.2 ± 7.4 |
| PANSS positive ± S.D. | - | 15.2 ± 4.7 |
| PANSS negative ± S.D. | - | 14.4 ± 6.1 |
| PANSS general ± S.D. | - | 30.8 ± 8.8 |
| PANSS total ± S.D. | - | 60.5 ± 16.7 |
| Chlorpromazine median dose (mg) (range) | - | 401.5 (67-2400) |

F: female, M: male, S.D.: standard deviation

**
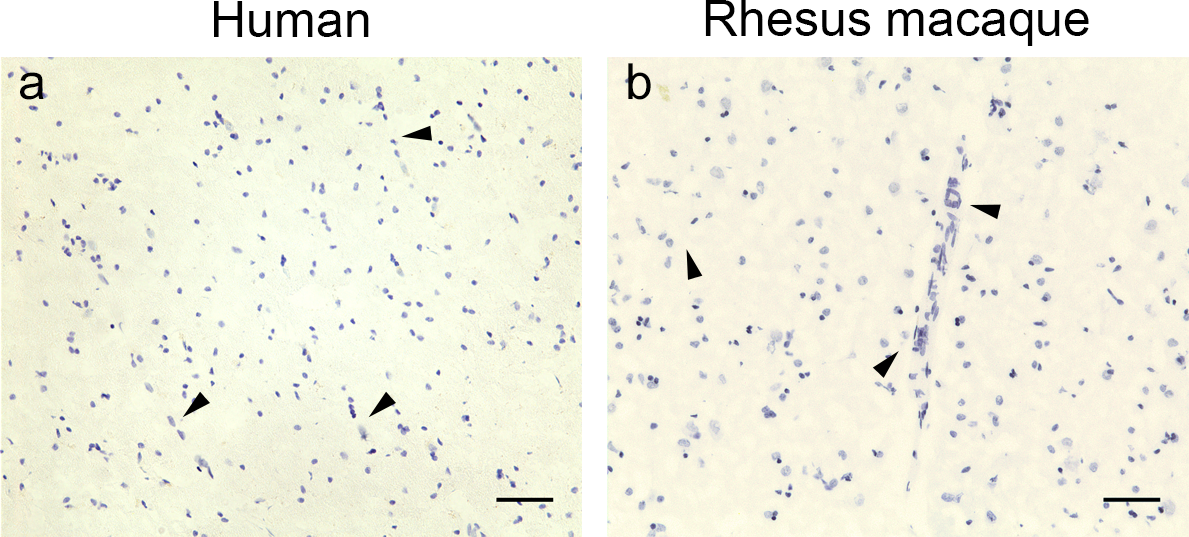
**

**Supplementary Figure S1:** DAB immunohistochemistry of human orbital frontal cortex no secondary control **(a)** and perfused Rhesus macaque cortex no primary control **(b)**. Nuclei are stained with a blue Nissl counterstain. Arrowheads indicate blood vessels. Scale bar is 50μm. Image taken with a 20x objective

**
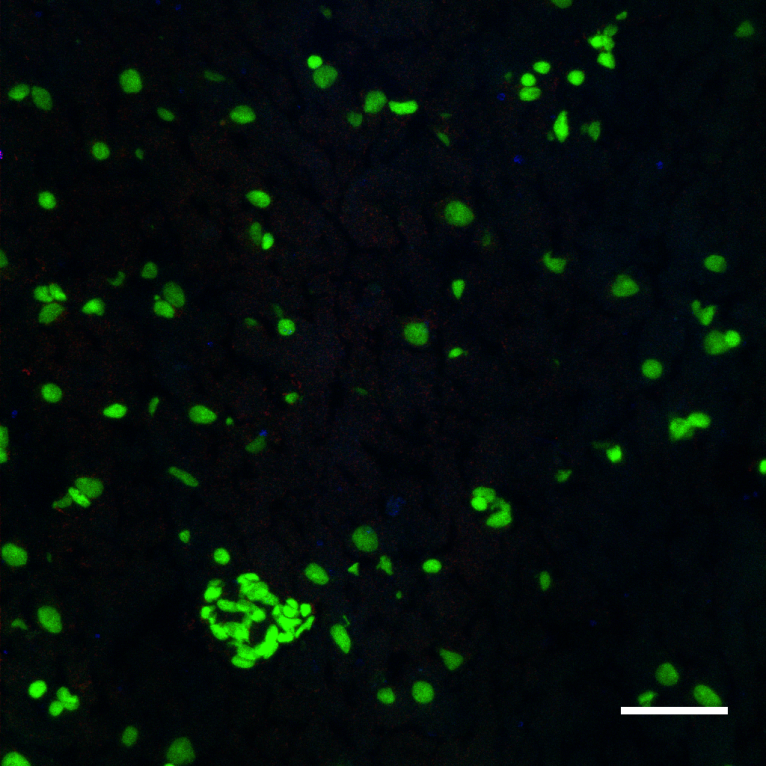
**

**Supplementary Figure S2:** No primary control slide triple label immunofluorescence of human orbital frontal cortex. Nuclei are stained green (488 nm) with an acridine orange counterstain. Scale bar is 50μm. Image taken with a 40x objective.

**
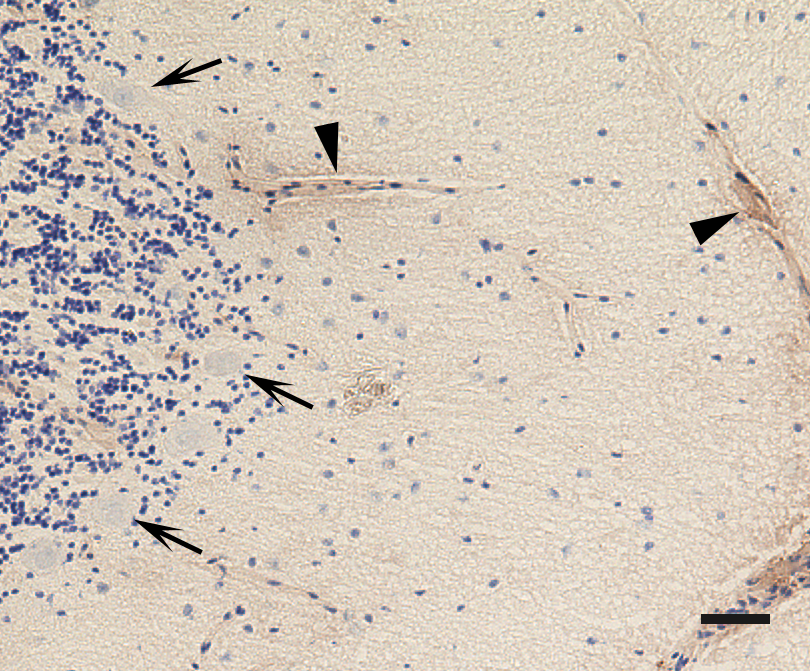
**

**Supplementary Figure S3:** Perfused adolescent rhesus macaque cerebellum no serum control slide. Nissl counterstained nuclei are blue. Staining of blood vessel is indicator of cross reactivity of anti-human antibody with endogenous IgG in the cerebellum. Fibrous staining considered background. Arrowheads indicate blood vessels. Arrows indicate Purkinje neurons. Scale bar is 50μm. Image taken with a 10x objective.


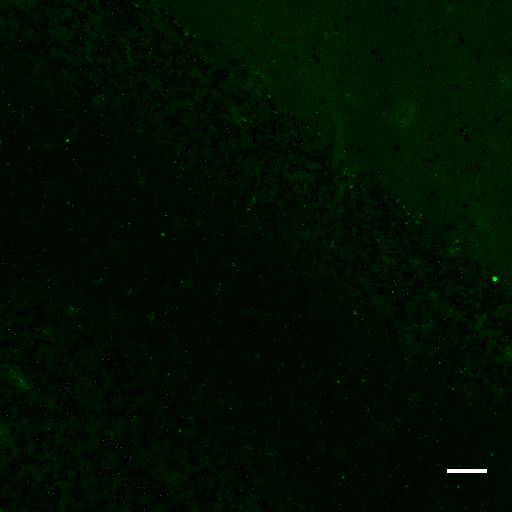


**Supplementary Figure S4:** Indirect immunofluorescence of primate cerebellum using manufacturers negative control solution (1:10 in phosphate buffered saline containing 0.1% Tween; EuroImmun AG, Lübeck, Germany). Scale bar is 50μm. Image taken with a 20x objective.

1. Fillman SG, Cloonan N, Catts VS, Miller LC, Wong J, McCrossin T*, et al*. Increased inflammatory markers identified in the dorsolateral prefrontal cortex of individuals with schizophrenia. *Mol Psychiatry* 2013; **18**(2)**:** 206-214.
